# Care for older forensic mental health patients: A consensus guidance document

**DOI:** 10.1192/j.eurpsy.2023.2413

**Published:** 2023-06-06

**Authors:** Jack Tomlin, Kate Walker, Jen Yates, Tom Dening, Kris Goethals, Birgit Völlm, Chris Griffiths

**Affiliations:** 1 School of Law and Criminology, University of Greenwich, London, UK; 2 Research & Innovation Northamptonshire Healthcare NHS Foundation Trust, Northampton, UK; 3 Mental Health & Clinical Neurosciences, School of Medicine, University of Nottingham, Nottingham, UK; 4 University Forensic Center, Department of Psychiatry, Campus University Hospital Antwerp, Antwerp, Belgium; 5 Department of Forensic Psychiatry, Rostock University Medical Centre, Rostock, Germany

**Keywords:** consensus guidance, forensic mental health, forensic psychiatry, gerontology, older patients

## Abstract

**Background:**

It is important to investigate the needs, experiences, and outcomes of older forensic mental health inpatients. In this consensus document, we offer practitioners working with older forensic inpatients recommendations to meet the unique older-age-related needs of this group.

**Method:**

We report on the findings of a scoping review of service provision and age-responsive interventions for this population. We complement this with a review of qualitative studies investigating staff and patient views on age-responsive inpatient care.

**Results:**

The guidance synthesizes this evidence into sections on: epidemiological studies of demographic, clinical, and legal profiles; qualitative studies; investigations of patient need; evidence for interventions tailored to this patient group; future directions for research; and finally, recommendations for practice. Forensic patients over the age of 50 years have a different set of psychological and physical health needs from their peers. There is a dearth of dedicated interventions and support to assist patients through secure services and into the community.

**Conclusions:**

We suggest service providers involve older patients in treatment and service organization decisions, adapt interventions to be responsive to this group, train staff to recognize physical vulnerabilities and cognitive decline, and embrace methods of communication developed in other areas of care, such as dementia Care.

## Background

In the United Kingdom (UK) and other Western countries, around 20% of inpatients in secure mental health settings are over 50 years old [1]. This percentage is likely to increase as people live longer and the older population proportion grows [2–4]. It is important to investigate the needs, experiences, and outcomes of these older forensic patients. This is because the age-related health needs of those considered “older” in the general population (typically around 60 years old) can be experienced by people with serious mental illness and individuals detained in the criminal justice system 10 years earlier [5]. The reasons for this disparity are myriad, encompassing poor access to healthcare, lifelong chronic illnesses, substance use backgrounds, and social deprivations like poverty and education and occupational opportunity.

The mental and physical health needs of older forensic mental health inpatients are multifaceted. They often have complex mental health histories, with high levels of psychotic disorders, personality disorders, and comorbidity [1]. Older adult forensic patients have histories that often include childhood neglect/abuse, substance abuse, poor self-management of health, cognitive difficulties, mobility problems, sensory impairment, psychiatric admission, and chronic physical illnesses (e.g., cardiac disease, high blood pressure, obesity, diabetes, hypertension) [6, 7].

Such complex needs mean that age-appropriate services are difficult to provide in secure mental health settings; the approach required is one that combines old-age psychiatric expertise and generic forensic psychiatry services. This means forensic services need to adapt regimes of care to accommodate this group. This might include developing specific services for older patients, making changes to the physical environment (e.g., including handrails that do not pose a ligature risk, wheelchair accesses), a particular focus on nurse–patient relationships, addressing physical mobility around units, and provision of somatic health care [1, 8]. Age-appropriate service provision involves balancing quality care in restrictive environments and supporting older forensic mental health patients to successfully move on from these settings, enabling them to access and maintain healthy lives when/if released from inpatient care. Not achieving this balance can lead to a life spent in secure care, institutionalization, homelessness, or poor health outcomes in the community, as older adult forensic mental health patients may be unable to adapt [9].

Best clinical practice supports the provision of specialized services for older patients in other fields of medicine (e.g., geriatric psychiatry, supported living). It, therefore, holds that similar efforts should be undertaken in forensic settings. Governments need to design innovative policies and public services specifically targeted to older persons [10].

## Rationale for this consensus guidance

Demographic shifts and the recognition of the need for age-appropriate forensic mental health services have co-occurred in recent decades. Research and guidance have lagged behind these developments. For example, a shared definition of “older” adult forensic mental health patients remains elusive. A recent review found little consistency in defining “older” (>50, >55, >60, etc.) [5]. There is no consensus whether older-aged, needs-based, or mixed-aged services enable the best recovery outcomes for this group [8]. Consequently, there is no specialized guidance and support for practitioners, policy-makers, or commissioners working with this older inpatient forensic population. Instead, care providers must apply best practices from a variety of sources.

## Aims and methods

This document aims to provide an overview of the evidence for mental health care for older forensic mental health inpatients and make recommendations to support service provision. It synthesizes recent research on the health profiles and needs of older forensic mental health inpatients. This guidance is divided into five sections. The first collates epidemiological studies describing the demographic, clinical, and legal profiles of older forensic mental health patients. The second describes qualitative studies investigating older forensic patients’ experiences of receiving mental health care in these settings. The third gives an overview of the evidence for interventions specifically for this patient group. The fourth considers future directions for research into older forensic mental health patients. Finally, recommendations that practitioners working with this group should consider are offered. This document complements the European Psychiatric Association (EPA) guidance on forensic psychiatry [11].

This consensus guidance is informed by a scoping review of the literature on interventions for older adult forensic mental health patients and a summary review of other published literature on this topic [12]. Articles were included where the study population comprised forensic patients (as inpatients or in the community) over 50 years who had experienced an intervention in the context of their care. A comprehensive range of search terms based on the following concepts were used: (older OR elderly) AND (“forensic mental health” OR “forensic patient”) AND (intervention OR treatment OR therapy). These were inputted into the following databases: PsychINFO, MEDLINE, CINAHL, EMBASE, AHMED, and the Psychology & Behavioral Sciences Collection. Google searches were also used and reference lists were hand-searched. This produced 2,572 results in total; 17 articles were subject to full-text review; and after excluding ineligible papers, eight articles were included in the review. Full details of the search can be found in Walker et al. [12]. Additional literature included in the present manuscript but not included in the scoping review was found by scanning the reference lists of the studies included in the Walker et al. review and searching Google Scholar for recent publications on the same population (until April 2023). Guidance for providing age-appropriate services was derived from this literature, clinical experience of the authors, and the results of empirical research conducted by the authors (the ENHANCE Study).

Percentages and frequencies reported in individual studies have been rounded to whole numbers to facilitate reading, unless specified.

## Epidemiological studies

Eleven papers reported on the findings from 10 studies. Studies describing the clinical, legal, and demographic characteristics of the older forensic population included in this section took place in the UK (*n* = 8), Canada (*n* = 1), and Germany (*n* = 1). One study was reported in two papers [13, 14]. Five adopted a retrospective cohort design, relying on clinical records [2, 7, 8, 15, 16]. Five conducted cross-sectional interviews [6, 13, 14, 17, 18]. Four compared older patients to their younger counterparts in the same services [2, 13–15]. “Old age” was defined as 45+ for indigenous patients [18], 50+ [6], and 50+ for nonindigenous patients [18], 55+ [7, 19], 60+ [2, 13, 17], and 65+ [16]. Girardi [15] did not define “old age,” instead grouping a cohort of patients aged 18 and older into six age categories (see Table 1). Natarajan and Mulvana [8] described an old-age ward without an explicit age threshold. We refer to older patients in this document as those described as “older” in these research papers by their authors, the youngest patient of which was 46. This is appropriate as we aim to summarize the extant evidence for this broadly defined population whose needs are influenced by age but not solely contingent upon age. Studies took place across low, medium, and high-security psychiatric inpatient settings (with the exception of Tomlin et al. [19] in which 27% of their sample were community patients).

**Table 1.** Characteristics of studies describing clinical, legal, and demographic characteristics of older forensic mental health patients

	Study design (inc. measures)	Country	Old age definition	Comparison group y/n	Sample size (total sample and % men)	Setting
Coid et al. 2002 [2] “Elderly patients admitted to secure forensic psychiatry services”	Retrospective, cohort, hospital records, 1988–1994	England	60+	Yes (16–59 vs. ≥60)	*N* = 52 (1.6%) of total admissions (*N* = 3,155); (94% men)	Medium and high security: 7/14 health regions
Tomar et al. 2005 [16] “Is there a case for a specialist forensic psychiatry service for the elderly?”	Retrospective, cohort of referrals, hospital records, 1990–2002	England	65+	Yes (age at first offense > or < 65 years)	*N* = 42 unique patients described in the study (90% men), from *N* = 78 referrals of patients ≥65 out of *N* = 5,477 total referrals (1.4%)	Medium security
Lightbody et al. 2010 [7] “A survey of older adult patients in special secure psychiatric care in Scotland from 1998 to 2007”	Retrospective, cohort, hospital records, 1998–2007	Scotland	55+	No	*N* = 36 (94% men) at admission or turned 55 during care	High security
Das et al. 2011 [14] “A comparative study of healthcare and placement needs amongst older forensic patients in a high secure versus medium/low secure hospital setting” and Das et al. 2012 [13] “Assessment of healthcare and placement needs in an older forensic psychiatric population in comparison to a younger forensic psychiatric population” (Both papers report findings on the same “older” patient sample)	Cross-sectional, interview, and hospital records; CANFOR-S, CANE-S, NABUS (forensic adaptation)	England	60+	Yes 2011: high versus medium/low security 2012: ≤ 45 versus ≥60	2011: *N* = 15 high versus *N* = 15 medium/low security. 2012: N = 26 ≤ 45; N = 30 ≥ 60 (100% men)	High, and medium-low security
Natarajan and Mulvana 2017 [8] “New horizons: Forensic mental health services for older people”	Retrospective; referrals over 18 month period; hospital records	England	Older adults or younger adults with early onset dementia or physical or mobility impairments	No	*N* = 25 referrals (100% men)	Dedicated older adult medium security ward
Girardi et al. 2018 [15] “Older adults in secure mental health care: health, social well-being, and security needs measured with HoNOS-secure across different age groups”	Retrospective, cohort, hospital records, 2007–2015; HoNOS-secure	England	No specific definition of “older”	Yes (18–24, 25–34, 35–44, 45–54, 55–64 and 65+)	*N* = 521 in total; 55–64: *N* = 32 (81% men); 65+: *N* = 24 (83% men) (55+ was 10.7% of the whole sample)	Low and medium-security hospitals
Di Lorito et al.2019 [6] “The characteristics and needs of older forensic psychiatric patients: a cross-sectional study in secure units within one UK regional service”	Cross-sectional, interview; hospital records; CANFOR-S, CANE-S (selected items), HCR-20, CAMCOG-R, PCL-R	England	50+	No	Phase One (records): *N* = 94 (89.4% men) (18.9% of total patient population)	Low, medium, and high-security hospitals
					Phase Two (interviews): *N* = 41 (44% of older population) (92.7% men)	
Tomlin et al. 2022 [19] “Older forensic mental healthcare patients in England: demographics, physical health, mental well-being, cognitive ability and quality of life [version 2; peer review: 2 approved]”	Cross-sectional interview, EQ-5D-5L, SWEMWBS, ReQoL, CCRT, MoCA, FRQ, hospitals records	England	55+	No	*N =* 37 (92% men)	Community, low, medium, and high-security hospitals
Stoliker et al. 2022 [18] “Older people in custody in a forensic psychiatric facility, prevalence of dementia, and community reintegration needs: an exploratory analysis”	Cross-sectional interviews; CSI’D′	Canada	45+ for indigenous patients (55%). 50+ for nonindigenous patients (45%).	No	*N* = 29 (96.6% men)	Regional forensic psychiatric hospital located in a medium-sized Canadian city
Verhülsdonk et al. 2023 [17] “Frequency of cognitive impairment in older forensic inpatients: results of a pilot cross-sectional study”	Cross-sectional interview; DemTect; MMSE; FAB; TMT A/TMT B; PQH	Germany	60+	No	*N* = 34 (100% men)	Five forensic psychiatric hospitals in North Rhine Westphalia

Abbreviations: CAMCOG-R, Cambridge Cognitive Examination-Revised; CANE-S, Camberwell Assessment of Needs in the Elderly – Short Version; CANFOR-S, Camberwell Assessment of Need, Forensic – Short Version; CCRT-SV, Cambridge Contextual Reading Test – Short Version; CSI’D′, Community Screening Instrument for Dementia; DemTect, Dementia Detection Test; FAB, Frontal Assessment Battery; FRQ, Forensic Restrictiveness Questionnaire; HCR-20, Historical, Clinical and Risk Management – 20; HoNOS-secure, Health of the Nation – Secure version; MMSE, Mini-Mental Status Examination German Adaptation; MoCA, Montreal Cognitive Assessment; NABUS, Nottingham Acute Bed Study questionnaire; PCL-R, Psychpathy Checklist – Revised; EQ-5D-5L; PQH-9, Patient Health Questionnaire; ReQoL, Recovering Quality of Life; SWEMWBS, Short Version Warwick-Edinburgh Mental Well-being Scale; TMT A/TMT B, Trail-Making Test A and B. Note: percentages are not rounded in this table to give an accurate representation of the number of women in these studies.

### Demographics and age

Men were the clear majority of patients in all studies and the proportion of women patients varied widely. No women patients were featured in the samples from [8, 13, 14, 17], but were respectively 19 and 17% of the 55–64 and 65+ groups in Girardi et al. [15], and 3% in Stoliker et al. [18]. This was largely due to the services included in these papers as studies did/did not recruit from sites with dedicated women’s services. Most older patients were single, separated, widowed, or divorced. The exception to this was the study by Coid et al. [2] who report only 31% of their sample as being single. The two studies describing ethnicity in the UK reported similar percentages of nonwhite patients: 12% [2], and 15% [6]. In Stoliker et al.’s [18] study based in Canada, 55% reported Indigenous status. Di Lorito et al. [6] also report patients’ religious affiliation: 54% Christian, 5% Muslim, 5% Buddhist, 2% Atheist, 7% “Other,” and 27% as “Undisclosed.” Verhülsdonk et al. [17] found that 47% had lower secondary education, 15% higher secondary, and 15% A-level equivalent diplomas, with the remaining having no graduation, attending a school for handicapped children, or having no data recorded on this point.

Compared to younger patients, older patients in Coid et al. [2] were significantly *less* likely to be single and nonwhite, and significantly *more* likely to be born outside the UK.

### Legal^1^

The sample from Coid et al. [2] had a mean age at first court appearance of 43 years. Most patients were reported as being in forensic care following criminal charges or convictions: 89% [2], 56% [7], 98% [6], and 83% [19]. Di Lorito et al.’s [6] sample was primarily detained following a hospital order (section 37/41 MHA, 1983; 42%) or prison transfer (section 47/49 MHA, 1983; 34%); this pattern was also found in Tomlin et al. [19]. In the study by Lightbody et al. [7], on admission, 39% of patients were on a hospital order with restrictions, 11% on a hospital order without restrictions, 17% were under assessment as a prisoner prior to sentencing, and 14% were under a civil section for medium to long-term treatment. Lightbody and others [7] reported that 42% of admissions were due to aggressive or disturbed behavior elsewhere. Coid et al. [2] found that 11% were not under a criminal section. One-third of the Tomar et al. [16] medium security sample was living in the community when the index offense was committed, one-third were in prison at the time of referral, 17% in high-security hospital, with 18% elsewhere. Most patients, but not all, across all studies had past criminal convictions. The most frequently reported index offenses were typically serious violent offenses; these are reported in Table 2.

**Table 2.** Most prevalent index offenses as reported in primary sources

Index offense	Percentage	Study
Homicide/Manslaughter	50	Coid et al. [2]
	25	Lightbody et al. [7]
	36	Natarajan and Mulvana [8]
	26	Tomar et al. [16]
	20	Verhülsdonk et al. [17]
(Attempted) Murder/Manslaughter	30	Tomlin et al. [19]
Attempted murder or grievous bodily harm	32	Coid et al. [2]
Violence against the person	21	Tomlin et al. [19]
Assault	39	Di Lorito et al. [6]
	12	Natarajan and Mulvana [8]
	6	Verhülsdonk et al. [17]
Aggravated bodily harm/threats	7	Coid et al. [2]
Threats to kill	8	Natarajan and Mulvana [8]
Arson	9	Coid et al. [2]
	9	Verhülsdonk et al. [17]
Sex offenses	6	Lightbody et al. [7]
	16	Natarajan and Mulvana [8]
	47	Tomar et al. [16]
	21	Tomlin et al. [19]
	57	Verhülsdonk et al. [17]
Acquisitive offenses	10	Tomar et al. [16]

*Note:* Percentages rounded to whole numbers. Index offences described as reported in primary sources.

Compared to younger patients, older patients were significantly *less* likely to have been convicted for “less serious” violent offenses like assault, threats to kill and robbery, and significantly *more* likely be older at time of first court appearance [2].

### Clinical

#### Mental health

Studies report a range of mental disorders, a summary of which is presented in Table 3. Recording, diagnosing, and reporting practices differed between studies, so Table 3 describes diagnoses as they were categorized by study authors (i.e., not aggregated into groups by the authors of this consensus guidance).

**Table 3.** Most prevalent diagnoses as reported in primary sources (≥10%)(either primary, secondary, or tertiary diagnosis)

Diagnosis	Percentage	Study
*Any schizophrenia, schizotypal and delusional disorders*	*20–64*	
Schizophrenia (inc. “Schizoaffective,” “Unspecified psychosis”)	33 (39)	Coid et al. [2]
Schizophrenia (inc. “Schizoaffective disorder” and “Other psychotic disorder”)	42 (48.8)	Di Lorito et al. [6]
Schizophrenia, schizotypal and delusional disorders	64	Lightbody et al. [7]
	60	Tomlin et al. [19]
	35	Verhülsdonk et al. [17]
Psychotic disorder	~31	Girardi et al. [15] (55–64)
	~20	Girardi et al. [15] (65+)
Schizophrenia or psychotic illness	24	Natarajan and Mulvana [8]
Schizophrenia/schizophrenia-like disorders	21	Tomar et al. [16]
Delusional disorder	29	Coid et al. [2]
*Any mood [affective] disorder*	*9–42*	
Mood disorders	14	Lightbody et al. [7]
Affective disorder	~9	Girardi et al. [15] (55–64)
	~37	Girardi et al. [15] (65+)
	16	Natarajan and Mulvana [8]
	19	Tomar et al. [16]
Mood [affective] disorders	16.2	Tomlin et al. [19]
Depression	42	Coid et al. [2]
Anxiety disorder	15	Di Lorito et al. [6]
Bipolar disorder	15	Di Lorito et al. [6]
*Any organic, including symptomatic, mental disorders*	*11–48*	
Dementia	48	Natarajan and Mulvana [8]
Organic brain syndrome	33	Coid et al. [2]
Organic disorders	21	Tomar et al. [16]
	11	Lightbody et al. [7]
*Any mental and behavioral disorders due to psychoactive substance use*	*14–62*	
Alcohol dependence/abuse	29	Coid et al. [2]
Substance Use/dependence	~50	Girardi et al. [15] (55–64)
	~62	Girardi et al. [15] (65+)
	15	Di Lorito et al. [6]
	14	Tomlin et al. [19]
Disturbance due to psychotropic substances	18	Verhülsdonk et al. [17]
*Any disorders of psychological development*		
Learning disability	10	Di Lorito et al. [6]
*Any disorders of adult personality and behavior*	*10–60*	
Personality and behavioral disorder	60	Di Lorito et al. [6]
	12	Natarajan and Mulvana [8]
	~19	Girardi et al. [15] (55–64)
	~24	Girardi et al. [15] (65+)
	42	Lightbody et al. [7]
	41	Tomlin et al. [19]
	53	Verhülsdonk et al. [17]
Antisocial	10 11	Coid et al. [2] Tomlin et al. [19]
Dissocial	14	Tomlin et al. [19]
Schizoid	10	Coid et al. [2]
Avoidant (anxious)	14	Tomlin et al. [19]
Emotionally unstable	11	Tomlin et al. [19]
Antisocial	11	Tomlin et al. [19]
Paranoid	11	Tomlin et al. [19]
*Other*		
Comorbid disorders (any)	54	Di Lorito et al. [6]
Paraphilia	56	Verhülsdonk et al. [17]
*Psychopathy*		
Psychopathy (>25 PCL-R)	8/14 (57) for whom PCL-R scores were available	Di Lorito et al. [6]

*Note:* ~ where data are reported graphically in primary source and exact figures are not provided. “Most prevalent” is defined as ≥10% when rounded. PCL-R, Psychopathy Checklist-Revised. Percentages rounded to whole numbers.

Lightbody et al. [7] examined clinical notes and found that 53% had previously self-harmed, 56% had harmful or dependent alcohol use, and 14% had previous substance abuse. These authors reported that 58% of patients had previous contact with forensic and 78% with general adult psychiatric services prior to their current placement. Of Di Lorito et al.’s [6] sample, 63% had never been admitted to secure forensic services before.

Di Lorito et al. [6] conducted cognitive assessments with their sample and found a mean cognitive assessment score of 86/100 (CAMCOG; excluding three outliers), with 21% scoring under the cut-off for normal cognitive functioning (80/100). For reference, CAMCOG general population norm values for men aged between 65 and 69 indicate that the median score is 92, with 5% of the population scoring 79 and below [20]. Verhülsdonk et al. [17] also describe cognitive ability in their sample: on the DemTect, 32% had results indicating mild cognitive impairment, 20% had suspected dementia, and 12% were unable to complete all tasks on the measure. 68% of the sample had cognitive impairment according to the Frontal Assessment Battery (FAB). The FAB-derived score correlated significantly with number of years of education (indicating higher education was linked to better cognition). Using the Mini-Mental State Examination (MMSE; German adaptation), they also found impairment in psychomotor speed (59%) and cognitive flexibility (59%) measured using the Trail-Making Test. Cognitive flexibility was significantly positively correlated with length of stay (higher MMSE scores indicate better cognitive functioning).

Compared to younger patients, older patients were significantly *less* likely to: have a diagnosis of schizophrenia or personality disorder (including ASPD and borderline PD) [2], have a history of drug and alcohol misuse [2, 13], and take antidepressants and mood stabilizers [13]. They were significantly *more* likely to: have lifetime diagnosis of delusional disorder, depression, and organic brain syndrome [2], a current diagnosis of schizoid personality disorder [2], and to be older at time of first admission to psychiatric hospital [2].

#### Somatic health

The studies suggest older patients have a high number of somatic conditions. Lightbody et al. [7] found that the average number of medical diagnoses on admission was 1.2, which rose to 2.4 at discharge or the end of the study period. Similarly, they found that the average number of medications at admission doubled (from 3.1 to 6.3). Tomlin et al. [19] report that on average, patients in their sample were prescribed 7.6 regularly taken medications and 2.1 psychotropic medications and had an average anticholinergic effect on cognition (AEC) score of 2.4. Note that AEC scores range from 0 to 3, with a lower score being desirable. A majority in Verhülsdonk et al.’s [17] sample were prescribed psychotropic medication (68%), specifically: neuroleptics (50%), cardiovascular medication (35%), antidepressants (27%), sedatives (21%) and antiepileptics (15%). Past alcohol abuse was reported in 77% of these cases.

Girardi et al. [15] reported that whilst around 25% of patients aged 55–64 had at least one physiological condition, 83% of those over 65 did. Di Lorito et al. [6] reported that 88% had at least one such condition. Of the patients examined by Natarajan and Mulvana [8], 76% had “significant” and 24% had “nonsignificant” physical health problems. Most patients (61%) in Lightbody et al. [7] had mobility problems and 19% had sensory impairment. Mobility problems were experienced by 28% of patients in Nataraja and Mulvana [8]. Di Lorito et al. [6] found the following illness prevalence rates: diabetes (27%), heart conditions (24%), high blood pressure (22%), obesity (22%), gastrointestinal system conditions (22%), musculoskeletal system conditions (22%), respiratory conditions (15%), and sensory impairment (10%). Tomlin et al. [19] report similarly high levels of somatic health burden: diabetes (49%), cardiovascular and circulatory system problems (38%), COPD (16%), visual impairment (14%), and asthma (11%) amongst others. These authors also found high average BMI scores in their sample; 32% were classified as “obesity class one” according to standards set by the World Health Organization [21]. Verhülsdonk et al. [17] found that 27% of their sample had a traumatic brain injury/accident and 12% apoplexy; they also report prevalence of hypertension (27%), diabetes (21%), and obesity (6%).

Compared to younger patients, older patients were significantly *more* likely to have eyesight, cardiovascular and endocrine problems [13].

#### Needs and risk

Studies reported that older patients generally have higher unmet needs than younger patients. The Camberwell Assessment of Need, Forensic – Short version (CANFOR-S), the HoNOS-secure, and the Camberwell Assessment of Need for the Elderly (CANE) tools were used for these comparisons. Using the HoNOS-secure, Girardi et al. [15] found that patients aged 55–64 showed no significant improvement between admission and discharge in the clinical domains: “severe disturbance,” “personal well-being,” “emotional well-being,” and “socio-economic status.” This was mostly true for the 65+ group who, however, did show significant improvements over time in “personal well-being.”

Of the older patients interviewed by Das et al. [13], one-third rated “treatment” as unmet on the CANFOR-S. Using the CANE, both patients and staff rated “physical health,” “memory,” “eyes/hearing/communication difficulties,” and “personal security” as unmet [13]. These authors found that older patients in high-security care had a higher number of unmet needs than those in medium/low security, with more of their needs being unmet regarding healthcare, psychological distress, basic education, and treatment [13]. Comparing the needs of older and younger patients, when total needs were compared, these authors found significantly more younger patients had met needs than the older patients [13]. They further noted that half the patients in high security would benefit from treatment outside of this level of security, whilst nearly all those in medium/low security needed low-security placements.

Di Lorito et al. [6] grouped their sample into 50–54 and 55+ age categories and found the former to have a higher number of unmet needs according to the CANFOR-S. The most met needs were in relation to: “eyesight, hearing, communication”, “treatment”, “information about condition/treatment”, and “food and money”. The most unmet needs concerned: “company”, “telephone”, “sexual expression”, and “daytime activities”. The authors report that the average Historical Clinical Risk Management-20, Version 3 (HCR-20, V3) score was 27/40, indicating medium risk. Further, 71% of patients had incidents of verbal or physical assault and 27% of self-harm or a suicide attempt in the past two years.

Stoliker et al. [18] asked staff to rate factors for discharge planning that they thought should be considered for older forensic patients on their caseloads. In order from most important, social workers and primary nurses both rated chronic illness, cognitive limitations, physical limitations, and mental health challenges as key needs to be addressed (percentage agreement on these factors ranged from 60 to 70%).

Compared to younger patients, older patients’ levels of assessed need generally remained static or improved less over time. In the study of Girardi et al. [15] using the HoNOS, the proportion of security items improving over time decreased as the authors examined older age groups. There were significant improvements in risk of harm to self in those aged 18–34 but not for those 35+; significant improvements in risk of harm from others in the 25–34 group only; risk of harm to others significantly improved in those aged 18–54 but not in those 55+; and finally, the need for risk management procedures significantly improved in those aged 18–24 but not in older groups. In the study by Das et al. [13] using the CANFOR, older patients were significantly *less* likely than younger patients to rate “alcohol-misuse”, “drug-misuse”, and “arson” as met needs. Younger patients were significantly *more* likely to rate “sexual expression” and “basic education” as unmet needs compared to older patients.

## Qualitative studies

Qualitative studies report broadly similar findings across settings and countries, lending credibility to the conclusions they draw. In many ways, participants’ narratives of their care do not differ markedly from those of younger patients reported in other studies [22]. Older patients discussed a lack of autonomy, the quality of food, understaffing, reduced activities, boredom, and uncertainty about the future, amongst other topics. This is informative as it tells us that the needs, experiences, and possible improvements to services for this older patient group overlap with those of their peers. Indeed, a recurring theme across these studies was that patient experiences were subjective and did not speak to a homogenous “older offender” voice [23]. Although the present section describes patients’ experiences of care, many of the themes can also be found in studies of staff perspectives [24]. The following sections describe three themes relevant to older offenders that emerged across the literature: (1) making sense of one’s place in the world, (2) daily living whilst in care, and (3) treatment and recovery needs.

### Making sense of one’s place in the world

Studies report older patients “making sense of their place in the world” in two key ways: (1) their identification with the label “old” and (2) how they situated their current “self” in the timeline of their lives. Visser et al. [25] reported a distinction made by patients between old age as maturity and wisdom, and old age as weakness and vulnerability. The former was associated with knowing one’s mental health and triggers and was considered positive. The latter led to a rejection of the “old age” label by some: “I am a youngster still (Nicholas, 50s)” (p. 3). The authors suggested that patients who viewed old age in these terms were less likely to seek assistance for physical health concerns, wanting to distance themselves from the “vulnerable” label. Jackson [26] reported patients wanting to be of value to others. For some, this could be achieved by using their experiences as older to offer advice to younger patients: “‘I think because I’ve been in so long I can give a bit of advice that makes them feel that wee bit better…I’m good at giving advice and they really appreciate it. And they’re all younger than me…so it’s good’ (P7)” (p. 75).

Most studies report that patients think about their present situation by referencing their past and future. Perspectives on the future or life after secure care were very different for each person. Some, cognisant of their age, were eager to move on, but the majority expressed anxiety at returning to the community. These concerns are related to feeling institutionalized, not finding work, moving into appropriate accommodation, or step-down facilities [23, 27]. For example: “‘No. It’s too late. By the time I’m out I’ll be…Too old by that time. Others want to get out but I don’t really want to, to tell the truth…’ (P8)” [26] (p. 77). Others conjured memories of their younger selves to distance their current self from when they offended, or to reminisce and rediscover old hobbies and skillsets to support their current recovery [26, 28].

### Daily living whilst in care

#### Practical and environmental features of care

Patients described several practical aspects of daily life that they felt should be adapted for older residents while in care. Units should be equipped with handrails and be wheelchair accessible [23]. Food ought to be more chewable (as well as varied and nutritious), delivered to older patients by others, and generally made more accessible: “They allow me not to queue for food. They bring it to my table (P02)” [29] (p. 123). Chairs can be located next to phones, so patients do not have to stand for extended periods [28]. Older patients need longer to shower than younger peers: “You should try it yourself: undressing, showering, drying and getting dressed within five minutes. This is really impossible” [27] (p. 977). Based on interviews with older patients [30], a common aspect desired by the patients was that they had within their environment their own bathroom facilities within their own rooms, and that these were not shared facilities. This afforded privacy and a preferred environment to reside within.

#### Activities

Views on activities were mixed. Some patients felt there were enough [25, 29]; others not [27]. However, dissatisfaction with the accessibility or meaningfulness of activities was widespread [23, 25, 26]. Patients felt too few activities catered for older individuals. Activities such as gardening, art, library visits, and watching sports were identified as more accessible. Patients expressed preference for activities aimed at older patients: “‘I’d be happier to see more people together in my age group in social functions or in the gym’ (P15; MS)” [28] (p. 943). Both patients and staff alike identified that if activities were meaningful, important to that person and in effect gave them a purpose day to day, this could facilitate better quality of life and progression for an older patient [24, 30]. These activities promoted feelings of being valued and respected and of engaging with something that was worthwhile. Some patients emphasized the value they saw in having friends and opportunities to meet friends within their hospital, meeting for coffee for example: “Yes. I have one, who I am very close to. We often drink coffee together. We discuss. Then, there’s another one who comes with me to the therapy. We are also close, but it’s different. It’s different because we’re together less often. Well we see each often, every day, but he likes to stay home. So, do I. We see each other anyway. (UF290).” [31] (p. 7).

#### Atmosphere

Although some patients appreciated mixed settings with more active younger patients, a preference for a settled atmosphere emerged in the studies. Patients worried about younger peers being aggressive or bullying older individuals [25, 28]. For example: “Most of the patients are younger than me, it can be a bit difficult when they are being childish or obstructive (Archie, patient)” [23] (p. 259). Walker et al. [24] found that conflicting dynamics arose between younger and older patients because of numerous differences identified such as in their outlooks, their tastes, music preferences, and the stages of lives that they were at. In their interviews some staff suggested that older patients were always in the minority and perhaps the “*odd ones out*” (S13, Psychologist). Yorston and Taylor [23] report patients wanting quiet areas; Visser et al. [25] and Jackson [26] found that patients favored routine. Indeed, some recalled detailed daily schedules, suggesting that institutional boundaries and restrictions supported the predictability of daily life. Some respondents wanted more frequent visits from family/friends. These visitors were often also older and found attending visits difficult due to security restrictions or travel distances [23, 28]. Patients in Verhülsdonk et al.’s [17] study of older forensic patients and prison residents, described life as isolating and lonely, attributing this in part to the opportunities, rules, and routines of the secure setting. Interestingly, the authors report that forensic settings seemed more facilitating of patient relationships than prison inmate relationships.

### Treatment and recovery needs

Studies confirmed that older patients have complex physical and mental health needs, some of which go unmet [23–25]. Most participants in De Smet et al. [27] had extensive histories of placements in penal or psychiatric settings. Patients in two studies identified psychological needs as especially problematic, expressing that they wanted more psychological support [27, 29]. Sexual needs were not often discussed, but Di Lorito et al. [29] reported these were important for some patients, but not everyone. Respondents in Jackson [26] felt that because older patients had often spent long periods of time in care, this group as well as staff were more familiar with individual risks, which was positive. Patients seemed more satisfied than not about the quality of physical healthcare [25, 28, 29]. One patient compared it to care in the community: “‘If I have pain in my back, they’d give me ibuprofen. When I was outside, there were times when I did not have ibuprofen in the medicine box in my flat’ (P40; LS)” [28] (p. 947). Walker et al. [12] found that it was identified in the narratives that older patients experience chronic illness, refractory illness, and serious disease. Common illnesses and diseases experienced by this population included: respiratory problems, diabetes, arthritis, angina and cardiac problems, COPD, and asthma.

Walker et al. [24] also reported that patients experienced a “hub and spoke approach to their care and recovery, where there was a core team around the patient (the hub) but also ready available access to other different professionals, services and support (the spokes) as and when patients need or require them” (p. 287). This included them having access to: a range of adjunctive health professionals and services, advocacy support services, alternative and complementary services, regular health checks and screening, and a multidisciplinary team of different professionals and experts.

Patients discussed their recovery journeys, including what aspects of mental health care they found most important. Most of the respondents in Yorston and Taylor [23] discussed moving on from their high secure setting, but very few spoke of moving to the community. Respondents in De Smet et al. [27] highlighted the lack of age-appropriate step-down services, rendering them stuck. Visser et al. [25] report patients being ambivalent about their next steps, reflecting a finding of Jackson [26] whose participants expressed little hope for the future. Within treatment, patients wanted more involvement in care decisions, and preferred psychosocial over psychopharmacological interventions [27]. Staff were generally seen in a positive light [25, 28, 29], but some respondents felt younger staff were not sensitive to the needs of older patients or lacked appropriate training to care for this group [25, 28]. Relationships with staff were especially important for this group given the amount of time spent together.

## Interventions for older forensic patients

For older patients in secure hospital units or in the community, generally there are no interventions developed specifically for them (i.e., older, forensic, and mental health), most are delivered across all ages, and to date, there is very little research that has examined and evaluated efficacy of the interventions that are currently offered for this group. Where there is limited research on interventions for older patients, this has been undertaken only in prisons [32], and this has not been extended to secure forensic hospital settings. Canada et al. [32] identified five unique interventions (within the seven papers included in their review) that targeted: depression (*BE-ACTIV*); physical, mental, and spiritual health (*TRU-GRIT*); trauma (*Art Expression*); communication and social skills (*Good Vibe*); and health/social care assessment and care planning (*Older Prisoner Health and Social Care Assessment and Plan*). Their review highlighted the different types of interventions (e.g., art and music therapy, group and individual counseling, recreational therapy, intensive assessment) that may be utilized in this population, and therefore potentially transferable to other forensic mental health settings. However, no conclusions could be drawn regarding the efficacy of these interventions, due to the lack of evidence (e.g., the absence of randomized controlled trials (RCTs)), control group comparisons, or measures of change overtime [32].

Another recent review [33] examined the evidence for the use of both psychological and psychosocial interventions offered to forensic mental health inpatients. However, this was not age specific, so while it would have included older patients; they were not examined as a discrete sample. Nine papers were included in the review. It was found that five broad types of intervention were offered to the patients: cognitive–behavioral therapy (CBT), dialectical behavior therapy (DBT), psychoeducation, schema-focused therapy (SFT), and solution-focused brief therapy (SFBT). They reported findings across a whole range of outcomes (such as quality of life, recovery, satisfaction, symptoms, violence, and risk), but only seven of the 91 comparisons analyzed were significant, and none of these significant findings revealed a consistent result. It was suggested that individual DBT and SFBT studies reported the most promising results. The authors concluded that the current evidence base for supporting any psychological or psychosocial intervention is limited.

The review by Walker et al. [12] found eight papers that were suitable for inclusion, four qualitative studies [25, 27, 28, 34] and four quantitative studies [6, 13, 14, 35]. None of these papers offered evaluations of interventions, or descriptions of specific interventions for older people.

The qualitative studies identified positive and negative perceptions of service provision. Regarding the former, De Smet et al. [27] found that patients appreciated the opportunity to participate in leisure and sport activities, having a sufficient range of therapies available, and receiving interventions with domiciliary follow-up when discharged into the community. Patients described some interventions as sufficient and useful, specifically voluntary and paid work, sports, cooking activities, and psycho-educational initiatives. Both Di Lorito et al. [28] and Visser et al. [25] reported that interventions and activities offered to older patients were suitable, well-received, age inclusive, and appropriate.

Studies described negative perceptions of service provision. For instance, patients identified that missing from service provision was help for alcohol misuse, appropriate psychological and psychiatric support, that there were not enough activities, that some activities provided were not useful and/or were age inappropriate and patients experienced boredom in relation to certain activities [27]. This held true across inpatients and community service provision. Long-term older patients experienced boredom due to the types and repetitiveness of interventions offered; they often had a lack of motivation to participate and engage [25, 28]. One factor that was found to be particularly problematic for older patients was because of their physical health issues and poor health, patients were unable to attend and/or physically unable to participate in certain interventions.

The quantitative studies discussed in the review were generally limited to the inclusion of descriptive data. Di Lorito et al. [34] offer some descriptives of, and a contextual understanding of, the types of interventions offered and experienced by those over 50 (e.g., art therapy, substance misuse therapy, music/dance therapy, violence reduction), suggesting that for some patients, certain activities did not meet older patients’ needs, although exactly which ones was not specified. Das et al. in two commentaries examined the health care and placement needs of older forensic mental patients across levels of security (high and medium/low) [14] and between older and younger patients [13]. As reported in section “Treatment and recovery needs”, needs were not being met for those in higher levels of security compared with lower levels, and for older patients in comparison with their younger counterparts.

The review by Walker et al. [12] concluded that a range of interventions and activities are available for all ages with none being identified as specifically developed for older patients. It was seen that for older patients there are appropriate individual activities that patients can choose to undertake such as cooking, physical activities, and voluntary work. However, it is not clear if there are more “formal” interventions (either one-to-one or in group format) that are designed for older forensic mental health patients to address specific outcomes of relevance to this population. Where formal interventions are available, these are offered across all ages; and as already noted, there is no research that has examined efficacy of these in older forensic mental health patients.

## Recommendations for research

Research focusing on older aged forensic patients is relatively recent. Most studies cited in this consensus guidance were published in the past decade. The consensus is that much more research is needed to address multiple knowledge gaps. Several topics have been highlighted by scholars as especially important for future investigation, including: needs assessment over time and how these differ from younger patients, barriers and facilitators to recovery, transitioning into the community, and the use of controlled clinical trial methods. These are addressed in more detail in this section.

Several authors reiterate that this group has a different clinical profile to younger patients and that a more comprehensive understanding of patient need is important [6, 7, 23]. To assess need, tools such as the HoNOS-secure, CANFOR-S, CANE, and DUNDRUM quartet can be used. Cross-sectional or longitudinal studies assessing patient needs would be most beneficial where different age groups are compared. In the future, addressing level and type of need might be a more useful way of thinking about specialized services that are not defined by the arbitrary chronological age threshold. The experiences and needs of women and ethnic minority patients should be explicitly included in these assessments [27, 29, 36].

More evidence is needed on barriers and facilitators to discharge and reintegration into the community. The range of additional hurdles faced by older patients is not clear but institutionalization, lack of adequate step-down facilities, and coordination between relevant services are potential barriers. De Smet et al. [27] suggest that formal liaison processes between old-age and forensic psychiatric services should be explored. Parrott et al. [36] call for research that explores patient experiences of transitions between services and how this relates to their level of optimism in care. Given that old-age care homes may not be able to manage the risk posed by some older forensic patients extending treatment in secure settings, Girardi et al. [15] suggest that future studies investigate whether length of stay and treatment progression is empirically linked with the risk of violence and index offense.

As the study by Girardi et al. [15] demonstrated, the speed and nature of recovery vary with patient age. Studies should ask what the most important aspects of recovery are for older patients and what barriers exist to achieving them. De Smet et al. [27] suggest comparing outcomes for older patients receiving care in a classical rehabilitation model (emphasizing security and risk management), to patients receiving care in a strength-based or recovery-oriented approach (e.g., the Good Lives Model (GLM): [37]). They further recommend exploring to what extent the domains of Self-Determination Theory (SDT; competence, autonomy/mastery, and relatedness) are targeted in treatment programmes. Similarly, Di Lorito et al. [29] propose that the Italian REMs model (small, community-based, low-security units) be given special attention when considering models of care for this group. Parrott et al. [36, 38] suggest that research be undertaken that explores diversion, sentencing, parole, and early-release discharge practices across the criminal justice system for older offenders with mental disorders.

In the review by Walker et al [12], a notable finding was the lack of research examining interventions for older forensic mental health patients. This means that too little is known about the effectiveness of interventions for older patients. There have been no RCT studies, quasi-experimental investigations, or even studies that examine change over time, to measure the efficacy of interventions. We have no evidence of what interventions are routinely offered and if they are effective across specific outcomes for this population. It is also not clear whether there are any interventions available that have been specifically designed for older patients as were seen to be available for older mental health patients in prison [32]. Scholars should specifically address or over-sample older patients when evaluating interventions (of any type) in forensic settings.

Finally, it should be noted that the quality of research is generally low to moderate. Sample sizes are typically small; data are often collected from hospital records, which are not always complete or accurate; and study designs are generally retrospective or observational. Databases recording routinely measured outcomes (routine outcome measures, ROMS; patient-rated outcomes measures, PROMS) for all patients can help here [39–42]. Future studies should be guided by existing models of well-being, recovery, or rehabilitation (such as SDT and GLM) or models adapted for older patients, so that theories and mechanisms of change can be empirically identified.

## Conclusion and recommendations for practice

Interest in providing specialized care for older forensic mental health patients has grown over the past two decades. It is becoming clear from research that older patients have a different set of psychological and physical health needs from their younger counterparts. It is also apparent that there is a dearth of dedicated interventions and support for older patients to assist them in their recovery through secure inpatient services and into the community. This consensus guidance has summarized the extant literature on the older forensic inpatient group, highlighting gaps in our knowledge and suggesting opportunities for future research.

This section synthesizes what is known about the older (over 50 years) forensic mental health inpatient patient group and proposes 31 recommendations for practitioners, researchers, and healthcare commissioners to consider when developing or improving services of older forensic patients. These recommendations were also included in the NIHR-funded ENHANCE study conducted by the authors of this consensus guidance document. This report presents independent research funded by the National Institute for Health Research (NIHR) under its Research for Patient Benefit (RfPB) Programme (Grant Reference Number PB-PG-1217-20028). The views expressed are those of the authors and not necessarily those of the NIHR or the Department of Health and Social Care.

### Value of forensic mental health services

1. Provide recognition of the contribution of inpatient and community forensic mental health services to older patients’ well-being and quality of life, as research suggests some older patients’ self-report assessment of well-being and quality of life might be similar to general population norms [19].

### Patient involvement in service provision

1. Take into account the views and preferences of older forensic mental health patients in service provision. This includes the built environment, access to meaningful activities, and plans for transition to other facilities or the community. Coproduction tools and resources should be applied.

### Service organization

1. Hospital/ward/unit rules, regulations, or routines should accommodate the needs of older patients.
2. Provide a comprehensive range of structured activities (chosen with patients’ input) to be undertaken in inpatient wards and the community, and offer at a range of participation and intensity levels.
3. Connect older patients to each other across multiple wards or facilities for activities and socializing, taking into account vulnerability and risk issues.
4. Provide activities that fit with patients’ interests and life course, that give them a sense of identity, purpose, and meaningfulness.
5. Adapt the physical environment to accommodate older patients’ needs and risks (e.g., mobility, sensory impairment, disabilities).
6. Provide healthy lifestyle choices: access to physical activities, exercise facilities, and healthy food options.
7. Staff levels and retention should be appropriately funded and adequate to support older patients’ needs, so that patient leave and going off the ward is possible and does not get canceled.
8. Enable patients to easily connect (face-to-face and via technology) with external family and friends and support new social connections, taking into account safeguarding.
9. Assess whether specific older adult interventions and services are required.

### Evidence-based care

1. Quality of life can be enhanced by addressing patients’ depression, cognitive impairment, anxiety, pain management, ability to perform usual (work, study, housework, self-care, social or leisure) activities, and mobility issues.
2. Quality of life and well-being can be enhanced by providing efficient and easy access to specialist healthcare services, including occupational therapists, physiotherapists, opticians, dentists, and dieticians.
3. Offer preventative assessments, medical screenings, and check-ups, and address issues identified appropriately and timely.
4. To reduce levels of obesity and diabetes, seek to improve patient physical activity levels, diet, and sleep quality.
5. Make allowances for cognitive impairments in needs assessment, risk assessment, interventions, and treatment.
6. Provide interventions and occupational therapy that supports cognitive functioning and functional abilities to enable people to live well and manage cognitive changes.
7. Provide evidence-based psychological interventions with options of group or one-to-one sessions.

### Transition and discharge

1. Offer suitable housing/supported accommodation in the community.
2. Provide consistent support and supervision throughout transition into the community.
3. Support access to appropriate meaningful work/activities/education for older patients to engage in after discharge.
4. Provide easy and fast accesses to community forensic mental health services so that patients have a safety net for support and to avert offending or a mental health crisis after discharge.

### Collaboration between different specialty groups

1. Develop strong links between healthcare services such as old age psychiatry, community forensic mental health teams, and somatic hospitals.
2. Develop strong links with organizations to support patients in the community (housing, social groups, charities, local authority, volunteering groups, and welfare support).
3. Develop collaborative innovation and research initiatives, conferences, webinars, and dedicated working groups within and across services.

### Staff training

1. Provide staff training in the care and treatment of older people with mental health problems; such as bereavement counseling, transitioning to the community, identifying indicators of dementia, and identifying predictors of mental disorder exacerbated by growing old in secure services, for example, loneliness, social isolation, and deaths of friends/family.
2. Provide staff training to support patients and their carers’ management of age-related health needs, such as cognitive difficulties, physical health conditions, mobility issues, sensory impairment, frailty, and incontinence.

### Language and communication

1. Eliminate stigmatizing language, labels, and stereotypical beliefs about older persons.
2. Communicate acceptance of who patients are now, rather than the person they were at admission or when they committed their index offense; acknowledge that patients and their risks change over time.
3. Provide information in a manner and format that reflects the range of cognitive abilities: adjusting vocabulary, grammar, imagery, spacing, pacing, text, font, and other communicative methods.
4. Ensure communication is a two-way process: patients’ voices are heard, and they are empowered to be part of decision-making processes.

## Footnotes

1 As most of these studies report findings from England and Wales, it is helpful to describe key features of the legal framework organizing forensic services in this jurisdiction. Treatment in secure (forensic) psychiatric hospitals is ordered under the Mental Health Act 1983 (as amended in 2007). This allows for both civil and forensic treatment orders. These orders are referred to as “sections.” Patients in secure psychiatric hospitals under a civil section are at a high risk of harm to themselves or others and the management of this risk cannot be appropriately achieved in general psychiatric services. The civil sections most relevant to secure settings are ss. 2 and 3 Mental Health Act 1983, for assessment and treatment respectively. Crucially, patients under a civil section have not been ordered into treatment following the commission of a crime; a conviction is not a prerequisite. Most patients are treated under forensic sections, which are ordered following the commission of a criminal offense. The most commonly used forensic sections under the Mental Health Act 1983 are as follows: s. 35 involuntary placement in a secure hospital for assessment during criminal proceedings; s. 36 involuntary placement in a secure hospital for treatment during criminal proceedings; s. 37 a hospital order for treatment of indefinite duration following conviction for an offense; s. 38 an interim hospital order for treatment following conviction where the court has not yet determined whether to make a hospital order or give a prison sentence; s 41 a restriction order that gives the Secretary of State final say over a patient’s discharge, transfer. and leave, instead of the responsible doctor or hospital management (this can be attached to a s. 37 hospital order); s. 45A a “hybrid order” in which courts can issue a prison sentence but order placement in a secure hospital at the start of this sentence, where a patient’s mental health sufficiently improves or treatment is considered to have no benefit then the patient will be transferred to prison to serve the remainder of their sentence; s. 47 the transfer of a prison inmate to a secure hospital for treatment after recognition of the necessity for mental health treatment (here, individuals can be transferred back to prison to complete their sentence or remain in hospital beyond their prison sentence where there persists a need for treatment); s. 48 individuals remanded in custody (“inmates”) who have not yet been sentenced to prison but require immediate mental health treatment in a secure setting; and s. 49 a restriction order for individuals transferred into hospital from prison (here again the Secretary of State has the final say regarding transfer, discharge, leave, etc., this is attached to s. 47).
